# A Wandering Abdominal Mass in a Neonate: An Enteric Duplication Cyst Mimicking an Ovarian Cyst

**DOI:** 10.1155/2017/9209126

**Published:** 2017-03-02

**Authors:** Shigeo Iijima

**Affiliations:** Department of Pediatrics, Hamamatsu University School of Medicine, Shizuoka, Japan

## Abstract

Enteric duplication cysts are rare congenital anomalies that are prenatally diagnosed through antenatal ultrasonography (US). In female patients, however, attention must be paid since these formations might be confused with ovarian cysts. Herein, we present a case of a low birth weight female infant with an enteric duplication cyst. A cystic lesion was detected in the right abdomen of the fetus on antenatal US and magnetic resonance imaging (MRI). Serial US and MRI examinations performed after birth showed a single cyst that wandered from side to side in the abdomen; the initial diagnosis was thought to be an ovarian cyst. During laparotomy, however, it was found to be an enteric duplication cyst with volvulus. To our knowledge, there has been no report of an enteric duplication cyst presenting as a wandering abdominal mass. Our experience indicates that early intervention is necessary for patients who have a wandering abdominal mass to avoid complications and urgent surgery, whether it is an ovarian cyst or an enteric duplication cyst.

## 1. Introduction

Enteric duplication cysts are rare congenital anomalies arising anywhere along the alimentary tract with a prevalence of 1/4500 autopsies [1]. Such cysts occur most commonly in the small intestine, and about half are in the mesenteric border of the ileum, sharing both a common muscular coat and arterial blood supply [2]. Enteric duplications may be suspected on sonographic demonstration of an intra-abdominal cystic mass in the second or third trimester of gestation. The differential diagnosis of antenatal intra-abdominal cysts includes ovarian cysts, renal cysts, choledochal cysts, hepatic cysts, mesenteric or omental cysts, and dilated bowel loops of intestinal atresia [3]. However, prenatal diagnosis of enteric duplication cysts is often difficult, and ultrasonography (US) identifies only 20 to 30% of lesions [4]. It is particularly difficult to differentiate an enteric duplication cyst from an ovarian cyst in a female fetus.

## 2. Case Summary

**Figure 1:**
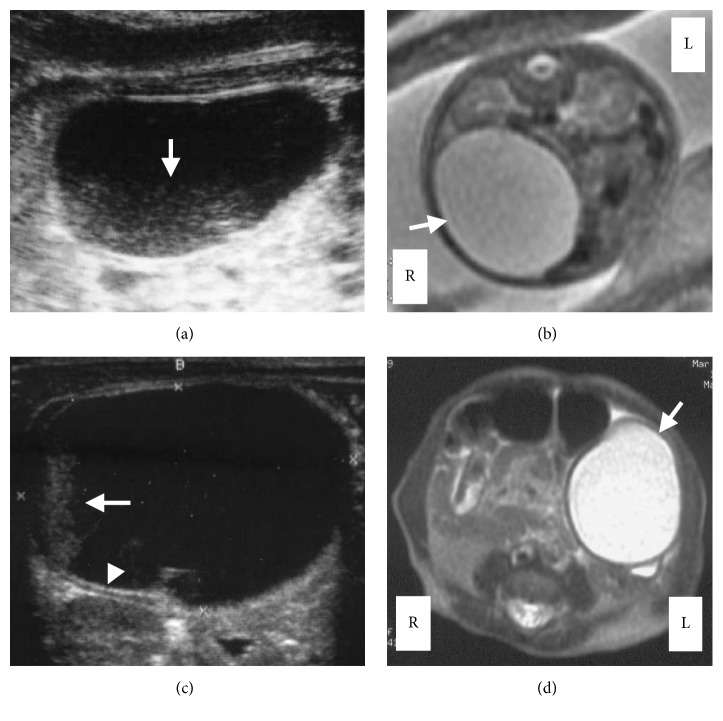
MR and US images of the abdominal mass. (a) Fetal ultrasonogram at 37 weeks' gestation: longitudinal section through the abdomen of the fetus shows a 4 × 3 cm cystic mass with sedimented echoes (arrow). (b) Fetal MR image at 28 weeks' gestation: axial T2-weighted image shows a cystic mass lesion in the right side of the fetal abdomen (arrow). (c) Postnatal longitudinal ultrasonogram on day 0 shows a 4.5 × 3.5 cm cystic mass with floating internal echoes in the right abdomen. Note the fluid-debris level (arrow) and muscular rim sign (arrowhead). (d) Axial T2-weighted MR image on postnatal day 16 reveals a hyperintense cyst in the left lower abdomen (arrow).

**Figure 2:**
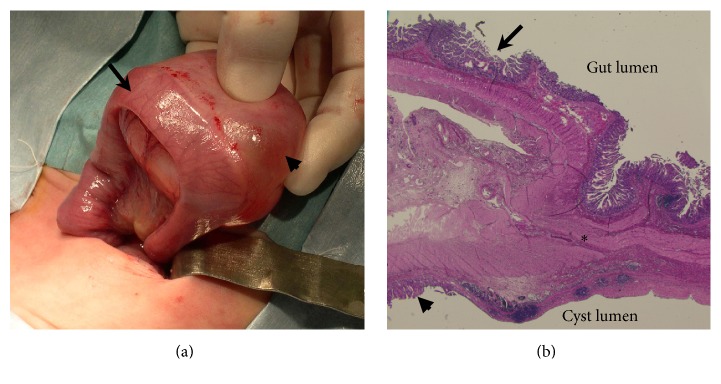
(a) Intraoperative photograph of the thick-walled, 5 × 5 cm cyst (arrowhead) attached to the ileum (arrow). (b) Low-power photomicrograph (hematoxylin-eosin stain; original magnification, ×100) shows histopathologic features of the enteric duplication cyst. The convergence of the cyst wall and the small-bowel wall can be seen. Duplication cyst mucosa of the duplication cyst including gastric mucosal lining (arrowhead) and mucosa of the native ileum (arrow) can be seen. The asterisk indicates the shared muscularis propria.

A fetus of 23 weeks' gestation was noted to have an intra-abdominal cystic mass that had been found during a routine prenatal US. This was the second pregnancy of a 27-year-old woman who, during her first pregnancy, had one normal neonate born by cesarean section (CS) because of breech presentation. The pregnant woman was referred to our hospital for evaluation of the fetal intra-abdominal mass. The growth of the fetus was slightly retarded for its gestational age with normal amniotic fluid volume. No other gross fetal abnormalities were identified. The US revealed a 4 × 3 cm unilocular cystic mass with sedimented echoes in the fetal right quadrant, and no significant thickness or hyperechogenicity of the cyst wall was seen (Figure 1(a)). A fetal magnetic resonance imaging (MRI), which was performed at 28 weeks' gestation, revealed a unilocular cystic structure without a thick wall and solid components occupying the right side of the fetal abdomen (Figure 1(b)). The radiologist suggested that the MRI findings made the diagnosis to likely be an ovarian cyst. The pregnancy was otherwise uncomplicated, and a 1950 g female infant was born at 38 weeks' gestation by a scheduled CS because of previous cesarean delivery. The neonate was admitted to the neonatal intensive care unit (NICU) and required supplemental oxygen because of mild respiratory distress. An initial physical examination showed a soft abdomen with no palpable masses. On admission, the US revealed a 4.5 × 3.5 cm cystic mass with floating internal echoes that projected into the right abdomen, just anterior to the right kidney. The US revealed that there was no evidence of communication between the mass and the intestine, and both ovaries were unremarkable. Based on the traditional US criteria, the differential diagnosis included ovarian cyst, bowel duplication, mesenteric cyst, and omental cyst. On US, echogenic debris and septation were seen in the cyst, and a double-layered wall was seen over a small segment of the lowermost portion of the cyst wall (Figure 1(c)), which revealed the transient change in contour of the cyst. To differentiate between the cyst and an enteric duplication and to determine whether the intra-cystic debris was a hemorrhage, an abdominal MRI was performed on day 16 of life. It showed a well-circumscribed cystic mass with a size of 3.8 × 3.5 × 3.0 cm in the left abdomen (Figure 1(d)). The cyst had a slightly thick and homogeneous wall and an incomplete septation-like structure inside. The cyst content showed hypointense signals on T1-weighted images and hyperintense signals on T2-weighted images. The MRI did not reveal any evidence of a hemorrhage in the cyst, an intestinal obstruction, or continuity with the wall of the intestine. The radiologist suggested that review of the MRI findings preferably revealed an ovarian cyst rather than an enteric duplication. Follow-up US studies on day 18 showed that the cyst had wandered to the right side of the abdomen. Therefore, we strongly suspected that the cyst was an ovarian cyst. Consultation with a pediatric surgeon regarding potential neonatal surgical management was done, and surgery was delayed until the neonate reached a satisfactory weight of more than 2500 g, as she was a low birth weight infant. Subsequently, US assessments at intervals of 3 to 4 days showed no changes in appearance, size, or location of the cyst. Additionally, the infant tolerated feeding and gained weight well. Toward the end of day 39 the infant suddenly became ill-tempered and developed abdominal distention and vomiting. Plain abdominal radiographs showed dilated intestinal loops. The infant was taken to surgery with a diagnosis of small-bowel obstruction caused by an ovarian cyst. On laparotomy, we observed a 5 × 5 × 3.5 cm cystic structure that was attached to the mesenteric border of the ileum, approximately 70 cm proximal to the ileocecal valve (Figure 2(a)). The cyst was spherical with a pinkish-tan, smooth, glistening surface. Mesenteric vessels extended over both surfaces of the duplication and supplied the duplication and the adjacent bowel. Moreover, volvulus of the ileum with a total counterclockwise rotation of 720° was observed. The cyst and the contiguous portion of the ileum were resected. Then, the volvulus was repaired and bowel continuity was restored by end-to-end anastomosis. During the surgery, the ovaries were not searched. When the cyst was opened, it was found to be unilocular and contained a clear, light-yellowish, mucinous fluid, including gelatinous material. A histological examination revealed that the resected cyst and contiguous portion of the ileum shared a common muscular wall, although each had its own mucosal lining (Figure 2(b)). This finding confirmed the diagnosis of a duplication cyst. The patient's postoperative course was uncomplicated. Oral feeding of the infant was restarted on the fourth postoperative day, and she was discharged 21 days later.

## 3. Discussion

Although it is difficult to distinguish duplication cysts from other types of cysts, the radiological appearance of these particular cysts has been described [2]. Before the availability of prenatal US, enteric duplications were likely to remain undetected unless the patient had signs and symptoms such as vomiting, abdominal distention, palpable abdominal mass, acute intestinal obstruction, intussusception, and volvulus. With improvements in the accuracy of prenatal US, cases of alimentary tract duplication have been identified in utero [5]. Variation in the ultrasonographic features of gastrointestinal duplications has been described; most lesions are observed as a cystic mass with internal debris, septations, and a peristaltic and double-layered (a hyperechoic inner layer and a hypoechoic outer layer) wall appearance that is compatible with the gastrointestinal tract wall [6]. In the present case, it was difficult to differentiate the abdominal cyst from an ovarian cyst, but postnatal US revealed characteristic findings of enteric duplication cysts. However, some problems can arise when establishing a diagnosis for an abdominal cyst. First, regarding the appearance of the cyst wall as seen on US, pitfalls in relying on the double-layered wall in the diagnosis of enteric duplication cysts have been reported [6]. However, an artifact that mimics the double-layered wall configuration of an enteric cyst has been described [7, 8]. A similar image can occasionally be found with an ovarian cyst [9]. Second, regarding the location of fetal abdominal cysts, it was reported that the visualization of a cyst in a high position does not always exclude an ovarian origin, especially if the cyst is large and may have extended to the upper abdomen from the pelvis [10]. Third, an MRI may be useful as a noninvasive technique for the diagnosis and accurate preoperative assessment of the cystic mass, and it is beneficial for the assessment of complications such as hemorrhages into ovarian cysts [11]. However, as for enteric duplication cysts, MRI findings may support sonographic findings but may give no additional information to those obtained using US when characterizing those cysts. Our case suggests that the diagnosis of duplication cysts using an MRI can be difficult, whereas the diagnosis by US can be suggested with confidence. Catania et al. reported that the most frequently prenatally misdiagnosed pathology was an enteric duplication while the highest rate of correct diagnosis was for a simple ovarian cyst [12]. In the present case, the most important diagnostic problem was that the 4 cm large cyst wandered from side to side in the abdomen on serial US and MRI examinations. The cyst was strongly suspected to be an ovarian cyst rather than an enteric cyst based on this finding, because ovarian cysts have been reported to be mobile, wandering around the abdomen on serial examinations [13]. However, the present cyst was an enteric duplication accompanied by volvulus. The duplication cyst, along with the adjacent contiguous bowel, had most likely wandered in the abdomen, resulting in volvulus. Extensive review of the literature failed to identify any other cases with enteric duplications describing a wandering abdominal mass. However, a duplication cyst may be displaced from side to side and be noticed as a wandering mass like an ovarian cyst, because enteric duplications are commonly located in the terminal ileum and are mobile. Therefore, in the differential diagnosis of an intra-abdominal cyst, the possibility of it being an enteric duplication cyst should be considered even if the mass wanders like an ovarian cyst.

Even if an enteric duplication is initially misdiagnosed as an ovarian cyst, and therefore treated conservatively, delays in making the correct diagnosis and treatment may not cause problems in asymptomatic patients. However, even when ovarian cysts are considered, a large and wandering cyst is believed to be at an increased risk of torsion [14]. On the other hand, enteric duplications can serve as the focal point for volvulus [4]. If the enteric duplication cyst is accompanied by volvulus, undiagnosed infants may experience life-threatening complications. Our experience indicates that a wandering duplication cyst may easily become symptomatic causing an intestinal obstruction. Therefore, to avoid complications and urgent surgery early intervention is necessary for those patients who have a wandering abdominal mass, whether it is an ovarian cyst or an enteric duplication. Unfortunately, we decided that an emergency surgery was not indicated for this case, and the elective surgery was delayed until the neonate reached a weight of more than 2500 g, as she was a low birth weight infant. This decision was made, because in Japan surgical outcome for premature babies weighing less than 2500 g at birth is still undesirable, although this has improved immensely for term babies of normal weight [15]. In case early surgical intervention is indicated in premature or low birth weight neonates, minimally invasive approaches are recommended for safety [16, 17]. Recently, it has become possible to treat neonatal surgical diseases in an effective and safe way with the laparoscopic technique [11, 18]. This technique is preferred for an undiagnosed complex cyst since it affords a diagnostic opportunity by direct observation of the lesion and an easier determination of its nature.

We first consulted a radiologist and then a pediatric surgeon to decide on management. However, we, pediatricians, should have discussed the diagnosis and management with an obstetrician, a pediatric surgeon, and a radiologist together, because multidisciplinary evaluation is crucial to establish a precise diagnosis and the management of neonatal patients with prenatally detected complex cysts.
